# Supplementary material for: Total Flavonoid Contents and the Expression of Flavonoid Biosynthetic Genes in Breadfruit (Artocarpus altilis) Scions Growing on Lakoocha (Artocarpus lakoocha) Rootstocks
Source: Plants (Basel). 2023 Sep 16;12(18):3285. doi: 10.3390/plants12183285 (PMC10534935; doi:10.3390/plants12183285)
Supplement: Supplementary file 1 [file plants-12-03285-s001.zip › suppl caption.pdf]

Figure S1 Representatives of breadfruit plants growing on different rootstocks.

Figure S2, Correlation of total flavonoid contents and *AaCHS* expression levels in breadfruit scions.

Figure S3, Representatives of the compatible grafts of breadfruit scions on lackoocha rootstocks. a) a graft showing scion section; b) a graft showing graft union region ; c) a whole grafted plant.

Table S1, Quantitative real-time PCR primers.
