# Supplementary figures and images for: Total Flavonoid Contents and the Expression of Flavonoid Biosynthetic Genes in Breadfruit (Artocarpus altilis) Scions Growing on Lakoocha (Artocarpus lakoocha) Rootstocks
Source: Plants (Basel). 2023 Sep 16;12(18):3285. doi: 10.3390/plants12183285 (PMC10534935; doi:10.3390/plants12183285)

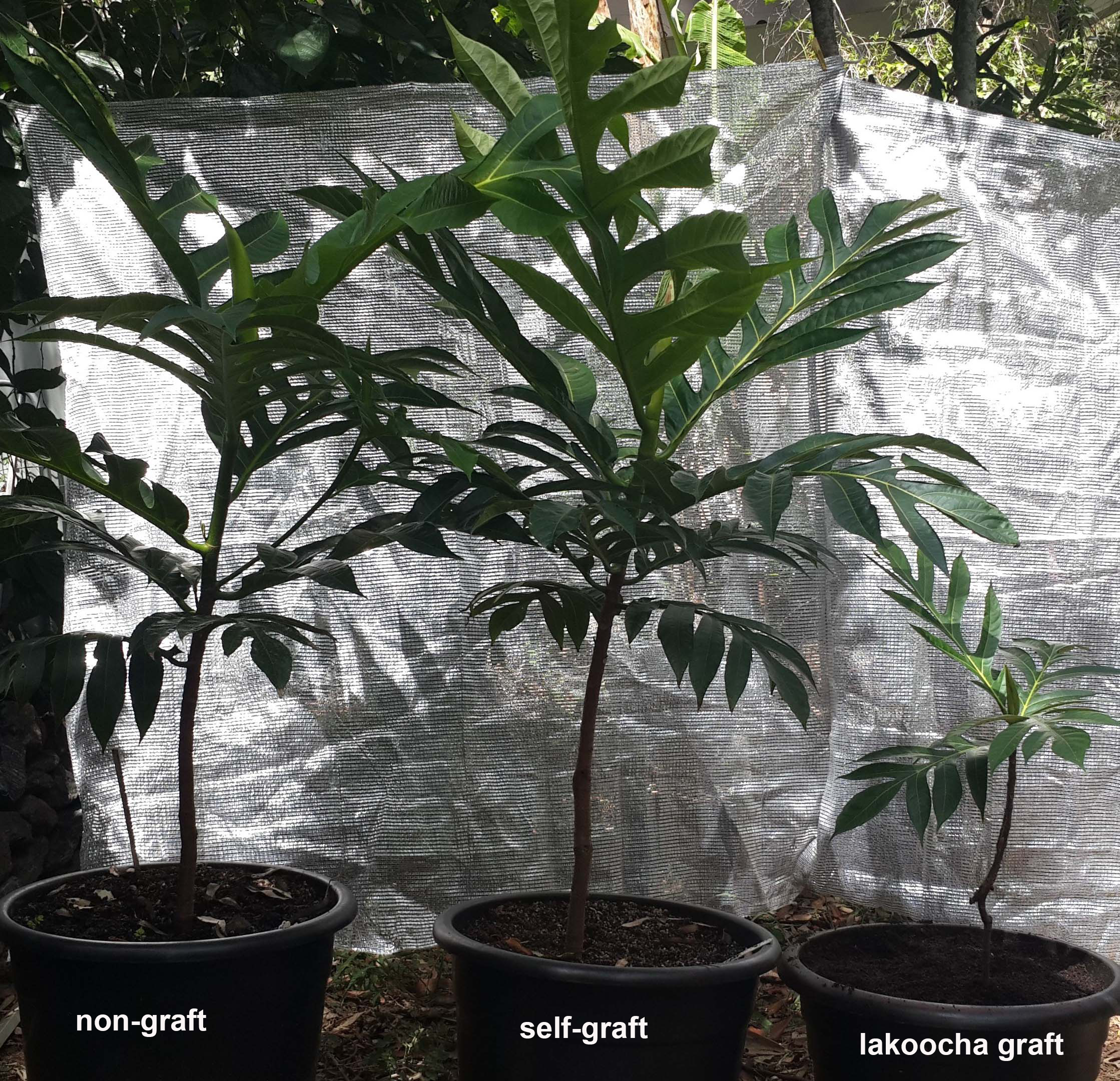

Supplement: Supplementary file 1 [file plants-12-03285-s001.zip › figure S1.tif]

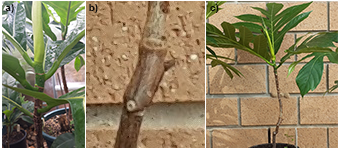

Supplement: Supplementary file 1 [file plants-12-03285-s001.zip › Figure S3.tif]
